# MiR-384 inhibits the proliferation of colorectal cancer by targeting AKT3

**DOI:** 10.1186/s12935-018-0628-6

**Published:** 2018-09-03

**Authors:** Yong-Xia Wang, Hui-Fang Zhu, Zhe-Ying Zhang, Feng Ren, Yu-Han Hu

**Affiliations:** Department of Pathology, School of Basic Medical Sciences, Xinxiang Medical University, Xinxiang, 453003 China

**Keywords:** Colorectal cancer, MiR-384, AKT3, Proliferation

## Abstract

**Background:**

Growing evidence suggests that MiRNAs play essential roles in the initiation and progression of colorectal cancer (CRC). The aberrant expression of miR-384 has been reported in some cancers. However, the role and mechanism of miR-384 in CRC proliferation remains unknown.

**Methods:**

The expression of miR-384 was detected in CRC and their paired normal tissues by real-time PCR. In vivo and in vitro assays were conducted to confirm the role of miR-384 in the proliferation of CRC. Bioinformatics analysis, luciferase reporter assays, western blot and in vitro assays were used to confirm that AKT3 was the target gene of miR-384. Finally, Spearman’s correlation analyses was carried out to analyze the relationship between miR-384 expression and AKT3 expression in CRC.

**Results:**

MiR-384 was down‑regulated in CRC tissues. The in vivo and vitro functional assays verified that the ectopic upregulation of miR-384 inhibited the proliferation of CRC and the inhibition of miR-384 promoted the proliferation of CRC. Bioinformatics analysis, luciferase reporter assays, western blot and in vitro functional assays confirmed AKT3 as the target gene of miR-384. The expression of miR-384 was negatively correlated with the expressions of AKT3.

**Conclusion:**

Our study verified that miR-384 could significantly suppress the proliferation of CRC by directing targeting AKT3.

**Electronic supplementary material:**

The online version of this article (10.1186/s12935-018-0628-6) contains supplementary material, which is available to authorized users.

## Background

Colorectal cancer (CRC) is one of the most common malignant tumors worldwide with high morbidity and mortality [1]. In the past 10 years, the incidence and mortality of CRC has increased rapidly in China [2, 3]. The initiation of CRC is a complicated process which includes the activation of oncogenes, the inactivation of tumor suppressor genes and multiple risk factors [4, 5]. Numerous genetic alterations, such as the microsatellite instability, PIK3CA, RAS and BRAF mutations, have been recognized to be involved in the tumorigenesis of CRC [6, 7]. However, other genetic and epigenetic alterations responsible for this disease remain largely unknown. Therefore, in-depth study of the molecular mechanism in the initiation and progression of CRC is significant for exploring novel molecular targets for CRC prevention and treatment.

MicroRNAs (miRNAs) are small non-coding RNA molecules which are highly conserved in evolution and participate in the regulation of gene expression by directly being bound to the 3′-untranslated region (3′-UTR) of their target mRNAs [8, 9]. It has been reported that miRNAs play crucial roles in the genesis and development of human cancer [10–12]. Recent studies have showed that miRNAs might be novel biomarkers for CRC. For example, MiR-215-5p expression is down-regulated in CRC [13]. MicroRNA-338-3p is down-regulated in thyroid cancer tissues and inhibits the progression of thyroid cancer by repressing AKT3 expression [14].

MiR-384 has been demonstrated to repress the proliferation and metastasis of pancreatic cancer [15]. Also, it has been indicated that miR-384 could inhibit CRC metastasis by directly targeting KRAS and CDC42 in our previous study [16]. The number of metastatic nodules in miR-384 over-expressed SW480 cells increased with the restoration of KRAS and CDC42. But the volume of the metastatic nodules could not be restored accordingly. The results suggested that miR-384 might regulate the proliferation of CRC not by targeting KRAS or CDC42. Therefore, in the current study, we will focus on delineating the role and mechanism of miR-384 in CRC proliferation.

## Methods

### Tissue specimens and cell culture

The fresh CRC and the matched normal colorectal tissues were collected from the Department of Pathology, Third Affiliated Hospital of Xinxiang Medical University (Xinxiang, China) from September 2016 to December 2017. All patients did not receive chemotherapy, radiotherapy or immunotherapy prior to surgery. All tissues were freshly frozen in liquid nitrogen until further use. All the cases had been diagnosed with adenocarcinoma on the basis of hematoxylin–eosin (HE) staining. The prior approval for the study had been obtained from Xinxiang Medical University Institutional Board (Xinxiang, China).

The stable cells of SW480/miR-384, SW480/Vector, HCT116/miR-384, HCT116/Vector, and LOVO/miR-384-in, LOVO/NC, SW620/miR-384-in, SW620/NC established in our previous study were cultured in RPMI-1640 or DMEM (Invitrogen). The cells of SW480, HCT116, LOVO and SW620 were obtained from American Type Culture Collection (ATCC). The medium was supplemented with 10% fetal bovine serum(FBS, Gibco) and 1% penicillin/streptomycin (Invitrogen).

### RNA isolation, reverse transcription (RT) and real-time PCR

Following the manufacturer’s instruction, the total RNA was extracted from the cultured cells and fresh CRC tissues with Trizol (Invitrogen, USA). 2 μg of total RNA synthesized the cDNA. Quantification of miR-384 was conducted by the All-in-One TM miRNA real-time PCR Detection Kit (GeneCopoeia, China) via the Applied Biosystems 7500 Sequence Detection system mixed with 5 ng cDNA and 10 pM of each primer. The cycling conditions were conducted as previously described [16]. As for the target gene of AKT3, the primers were shown in Additional file 1: Table S1. The data were normalized to U6 or GAPDH and calculated as 2^−ΔΔCT^.

### Western blot

Protein lysates obtained from the cells were subjected to SDS-PAGE and then the gel was transferred to Polyvinylidene difluoride (PVDF, Merck Millipore). The PVDF membranes were blocked with 5% non-fat dry milk and then the membranes were incubated with rabbit anti-AKT3 (1:200, Proteintech, USA) or mouse anti-α-tubulin (1:2000, Proteintech, USA) overnight at 4 °C. The next day, they were incubated with the appropriate secondary antibodies (HRP-conjugated anti-rabbit IgG (1:5000, CST, USA) or HRP-conjugated anti-mouse IgG (1:5000, CST,USA) and detected with a chemiluminescence imaging analysis system (Tanon, China).

### MTT assay

1 × 10^3^ cells were seeded on 96-well plates and cultured for 24 h. 20 μl 5 g/l 3-(4,5-dimethylthiazol-z-yl)-2,5-diphenyltetrazolium bromide (MTT, Sigma, USA) was added to each well and incubated for 4 h. Then, MTT was removed and 150 μl dimethyl sulphoxide (DMSO; Sigma, USA) was added to the wells. The absorbance was measured at 450 nm with a microplate autoreader (Bio-Rad, Hercules, CA, USA). The experiment was conducted repeatedly for three times.

### Colony formation assay

The cells were trypsinized and plated on 6-well plates (200 cells/well) and cultured for 2 weeks. The colonies were stained with Hematoxylin for 30 min after fixation with 4% paraformaldehyde for 10 min. The number of colonies, defined as > 50 cells/colony, was counted. Three independent experiments were performed.

### Soft agar assay

Six-well plates were covered with a layer of 0.6% agar (Sigma, USA) in medium supplemented with 20% fetal bovine serum. Cells were prepared in 0.3% agar and seeded in triplicate at 3 a dilution of 1 × 10^3^. The plates were incubated at 37 °C in a humid atmosphere of 5% CO_2_ for 2 weeks. Each experiment was repeated at least 3 times. Colonies were photographed after 2 weeks at an original magnification of 200×.

### Tumorigenesis in nude mice

4 to 6-week-old BABL/c nude mice were purchased from the Center of Laboratory Animal Science of Guangdong (Guangzhou, China). All animal experiments were conducted in accordance with current Chinese regulations and standards regarding the use of laboratory animals, and all animal procedures were approved by the Xinxiang Medical University Institutional Animal Care and Use Committee. Xenograft tumors were generated by subcutaneous injection of 2 × 10^6^ stable cells of SW480/Vector, SW480/miR-384, LOVO/NC, and LOVO/miR-384-in (n = 6) on the hindlimbs. Another 6 nude mice were used to conduct the restoration experiments in vivo. 3 weeks later, all mice were euthanized by dislocating the cervical spine. Tumor size was measured by a slide caliper (volume = length × width × height). The tumors were fixed with 10% neutral buffered formalin and embedded in paraffin. Then 4 μm sections were cut and stained with haematoxylin and eosin according to standard protocols. Sections further underwent immunohistochemistry (IHC) staining: They were sections baked at 60 °C for 1 h, deparaffinized with xylenes and rehydrated with graded ethanol. After incubation in 3% H_2_O_2_ to quench the endogenous peroxidase activity, the sections were heated in 0.01 M, sodium citrate buffer, pH6.0, for antigenic retrieval. Later, the sections were blocked with normal non-immume serum for 20 min and then incubated by mouse anti-Ki-67(Maixin, China) overnight at 4 °C. The next day, the sections were treated with secondary antibody followed by streptavidin–horseradish peroxidase complex. Finally, DAB was used for colour development. PBS was used to replace the primary antibody as the negative control. Finally, the stained slides were evaluated independently by two pathologists who were blind to the clinical parameters. The positive tumor cells were stained for Ki-67 protein in the nucleus.

### Plasmid construction, transfection and luciferase assays

The miR-384 binding site in the AKT3 is located at 4362–4367 bp, whose full length of 3′UTR is 5439 bp. The region of human AKT3 3′UTR at 4141–4510 was PCR-amplified and inverted into the *Xho*I/*Not*I sites of the psiCHECK-2 luciferase reporter plasmid (Promega). The primers were as follows: F: CCGCTCGAGATACACGCAAATACACTCC; R: GGGGCGGCCGCCTTCTACAGTATCCACCAC. Cells were seeded in 24-well plates (1 × 10^5^/well) the day before transfection. Then the psiCHECK-2-luciferase reporter gene plasmids psiCHECK-2-AKT3-3′-UTR or control-luciferase plasmid were transfected into the cells with the control pRL-TK Renilla plasmid (Promega) by Lipofectamine 2000 Reagent (Invitrogen). Luciferase and Renilla activities were assayed 48 h after transfection by the Dual Luciferase Reporter Assay Kit (Promega) following the manufacturer’s instructions. All experiments were conducted at least 3 times and the data were presented as mean ± standard deviation (mean ± SD).

### Statistical analysis

All statistical analyses were carried out by SPSS20.0 for Windows. The data were expressed as the means ± standard deviations (SD) from at least three independent experiments. The comparisons of the means were carried out by one-way analysis of variance (ANOVA) test with post hoc contrasts by LSD test. Comparisons were considered significant when p < 0.05. The comparison was conducted by Mann–Whitney U-test. Spearman’s correlation analysis was carried out to analyze the relationship between miR-384 expression and AKT3 expression. Statistically significant data were indicated by asterisks: *p < 0.05 or **p < 0.01.

## Results

### MiR-384 was down-regulated in CRC tissues

**Fig. 1:**
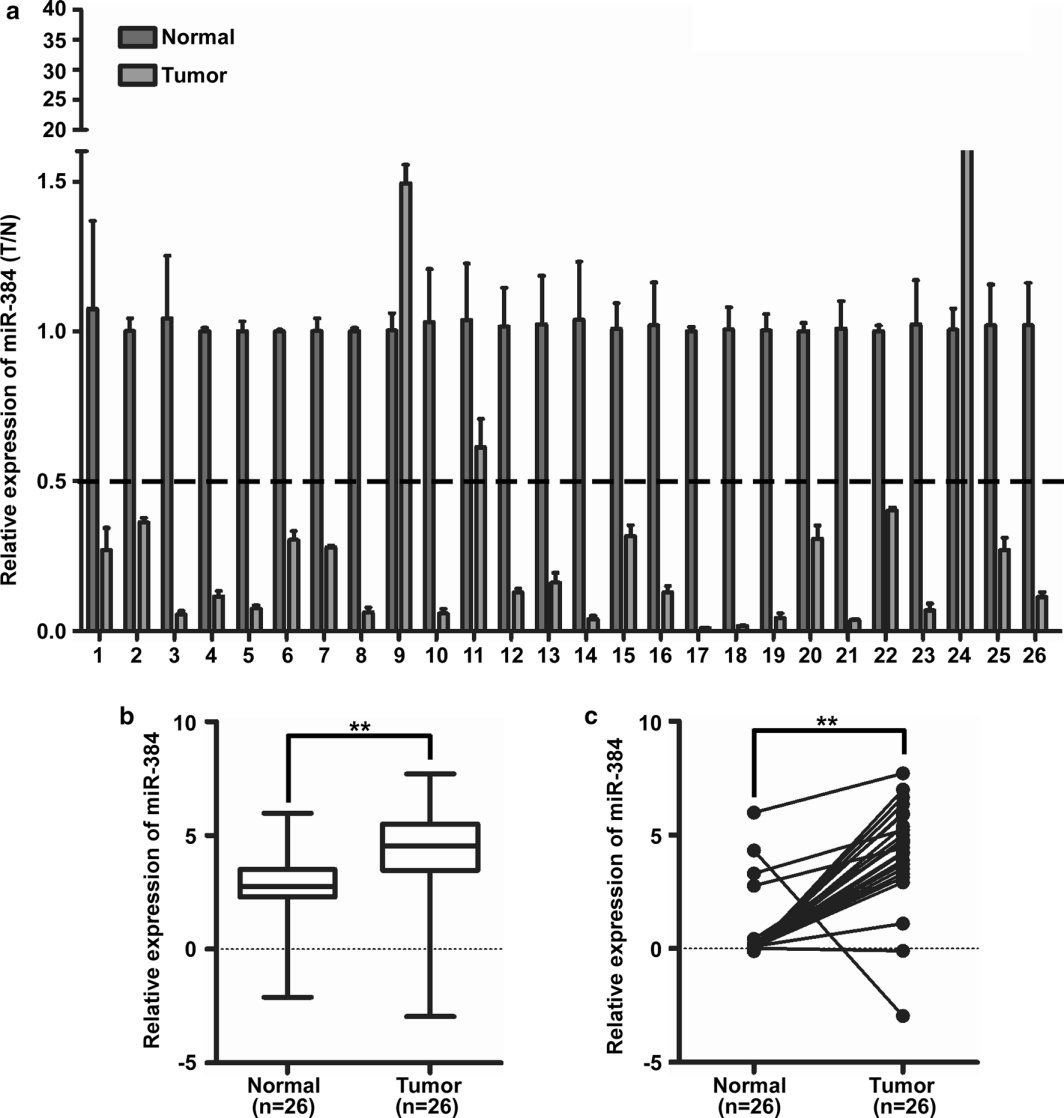
MiR-384 was down-regulated in CRC tissues. **a** Expression of miR-384 in 26 cases of fresh human CRC tissues and their matched adjacent normal tissues by real-time PCR analysis; miR-384 expression was normalized to U6 and expressed relative to the matched adjacent normal tissues (2^−ΔΔCт^). **b**, **c** Mean expression of miR-384 in 26 cases of fresh human CRC tissues and their matched adjacent normal tissues by real-time PCR (ΔCт, n = 26, **p < 0.01), boundaries of boxes represent bounding of the boxes and stand for the lower and upper quartile. Lines within the boxes and whiskers represent median and extremum (maximum and minimum)

The expression of miR-384 was detected by qPCR analysis in 26 cases of CRC biopsies and their matched normal samples obtained from the Third affiliated Hospital. It was found that miR-384 was down-regulated in 88.5% (23/26) of CRC tissue samples (T) compared to their matched adjacent normal tissues (N) (Fig. 1a). Student’s *t* test showed that the expression level of miR-384 in CRC tissues samples was significantly lower than that in adjacent normal tissues (Fig. 1b, c). Taken together, these results showed that miR-384 was down-regulated in CRC tissues.

### Over-expression of miR-384 inhibited the proliferation of CRC cells in vitro and in vivo

**Fig. 2:**
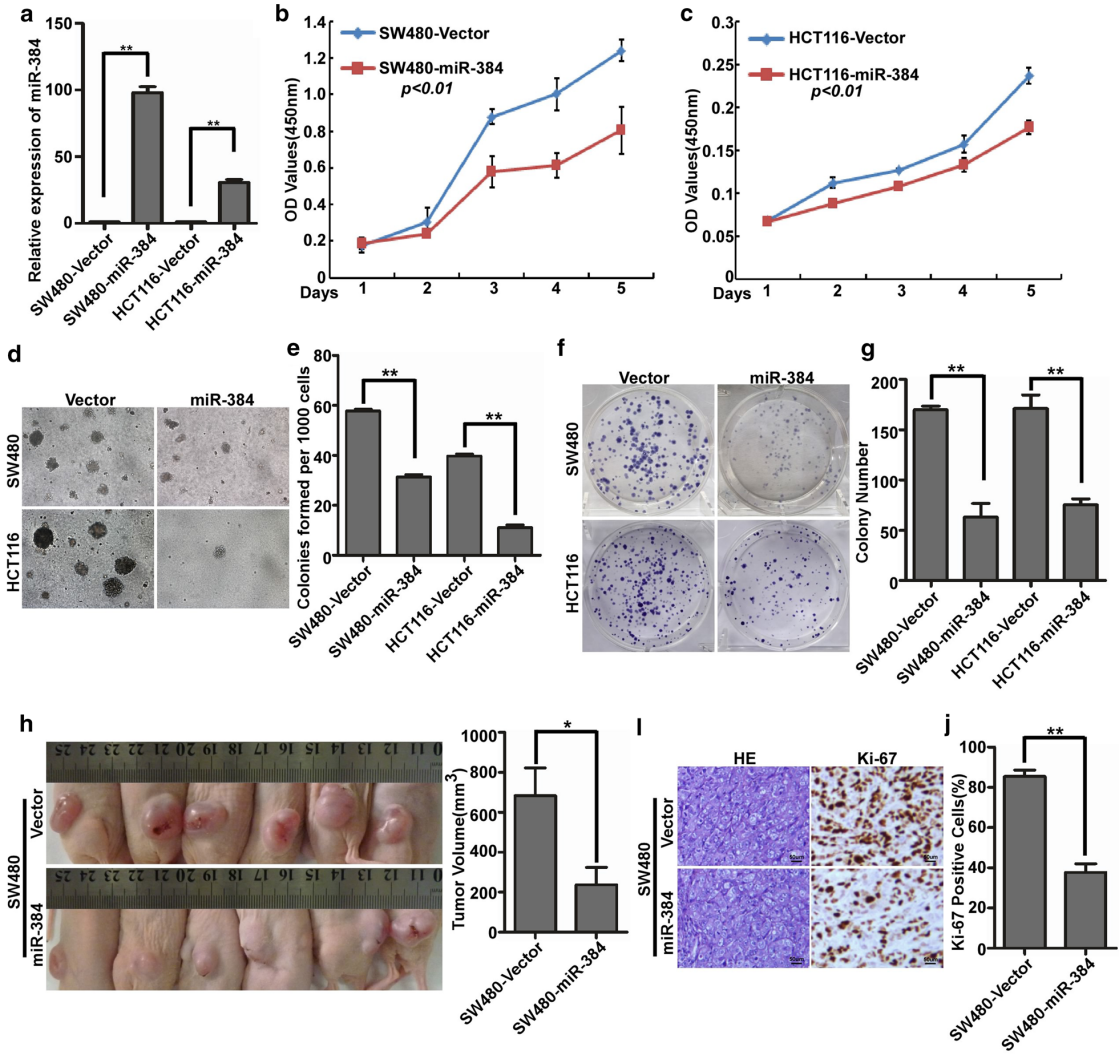
Over-expression of miR-384 inhibited the proliferation of CRC cells in vitro and in vivo. **a** Over-expression of miR-384 in SW480 and HCT116 CRC cells verified by real-time PCR. **b**–**g** The proliferative ability of the indicated cells detected by MTT assays, colony formation assays and soft agar assays. Only cell colonies containing more than 50 cells were counted. Error bars represent mean ± SD from 3 independent experiments. **p < 0.01. **h** SW480/miR-384 and SW480/Vector cells were injected into the hind limbs of nude mice (n = 6). The tumor volume data were presented as the mean ± SD. **i**, **j** Histopathological analyses of xenograft tumours. The tumor sections were stained with H&E or subjected to IHC staining using an antibody against Ki-67. Error bars represent mean ± SD from three independent experiments. **p < 0.01

To explore the possible role of miR-384 on the proliferation of CRC, MTT, soft agar and colony formation assay were performed with the stable cells of SW480/miR-384, SW480/Vector, HCT116/miR-384 and HCT116/Vector (Fig. 2a). The results showed that the over-expression of miR-384 obviously decreased the OD value of MTT, the colony number of soft agar and colony formation assay of SW480 and HCT116 (Fig. 2b–g). So, miR-384 overexpression significantly inhibited the proliferation of CRC cells in vitro. To further confirm the effect of miR-384 in inhibiting the proliferation of CRC cells in vivo, we performed the tumorigenesis assay in nude mice and found that the volume of tumors in SW480/miR-384 group was much smaller than that in SW480/Vector group (Fig. 2h). IHC confirmed that the tumors of the SW480/miR-384 group showed much lower Ki-67 indices than that in control group (Fig. 2i, j).

### Inhibition of endogenous miR-384 promoted the proliferation of CRC cells in vitro and in vivo

**Fig. 3:**
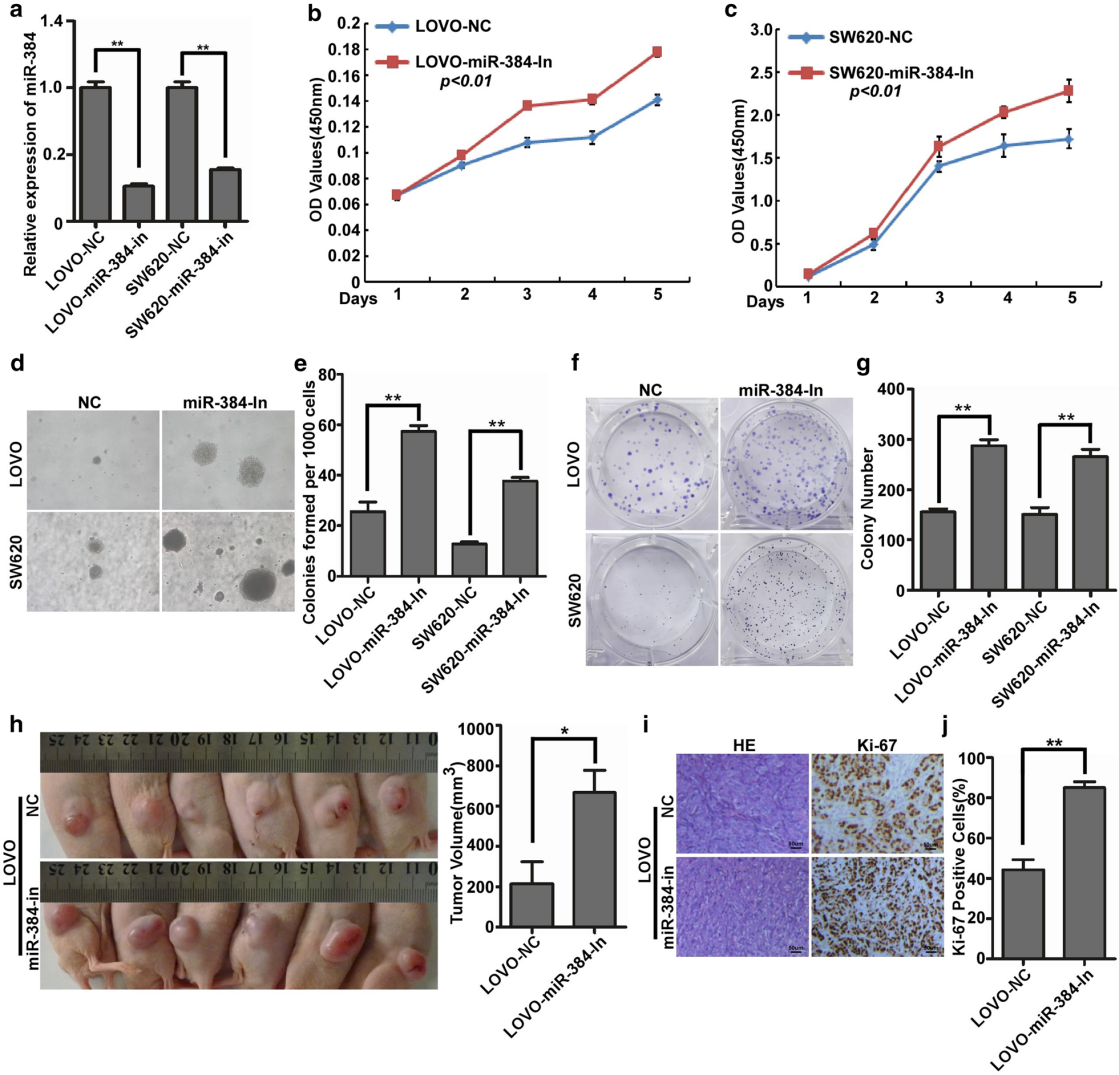
Inhibition of endogenous miR-384 promoted the proliferation of CRC cells in vitro and in vivo. **a** Expression of miR-384 in LOVO and SW620 CRC cells transfected with inhibitor or their paired negative control lentiviral vector was detected by real-time PCR. **b**–**g** The proliferative ability of the indicated cells detected by MTT assays, colony formation assays and soft agar assays. Only cell colonies containing more than 50 cells were counted. Error bars represent mean ± SD from 3 independent experiments. **p < 0.01. **e** LOVO/miR-384-in and LOVO/NC cells were injected into the hind limbs of nude mice (n = 6). The tumor volume data were presented as the mean ± SD. **i**, **j** Histopathological analyses of xenograft tumours. The tumor sections were stained with H&E or subjected to IHC staining using an antibody against Ki-67. Error bars represent mean ± SD from three independent experiments. **p < 0.01

The proliferative abilities of the stable cells LOVO/miR-384-in, LOVO/NC, SW620/miR-384-in and SW620/NC were detected by MTT, soft agar and colony formation assay (Fig. 3a). The results demonstrated that the suppression of miR-384 obviously increased the OD value of MTT, the colony number of soft agar and colony formation assay of LOVO and SW620 cells compared with their negative control group (Fig. 3b–g). Therefore, miR-384 knock-down significantly increased the proliferative abilities of CRC cells in vitro. To further observe the inhibition effects of miR-384 on CRC proliferation in vivo, LOVO/miR-384-in cells and the control cells LOVO/NC were injected to the hind limbs of the nude mice. Results demonstrated that LOVO/miR-384-in group showed much lager tumors and much higher Ki-67 indices than that in LOVO/NC group (Fig. 3h–j).

### MiR-384 decreased AKT3 expression by directly binding to it’s 3′UTR in CRC cells

**Fig. 4:**
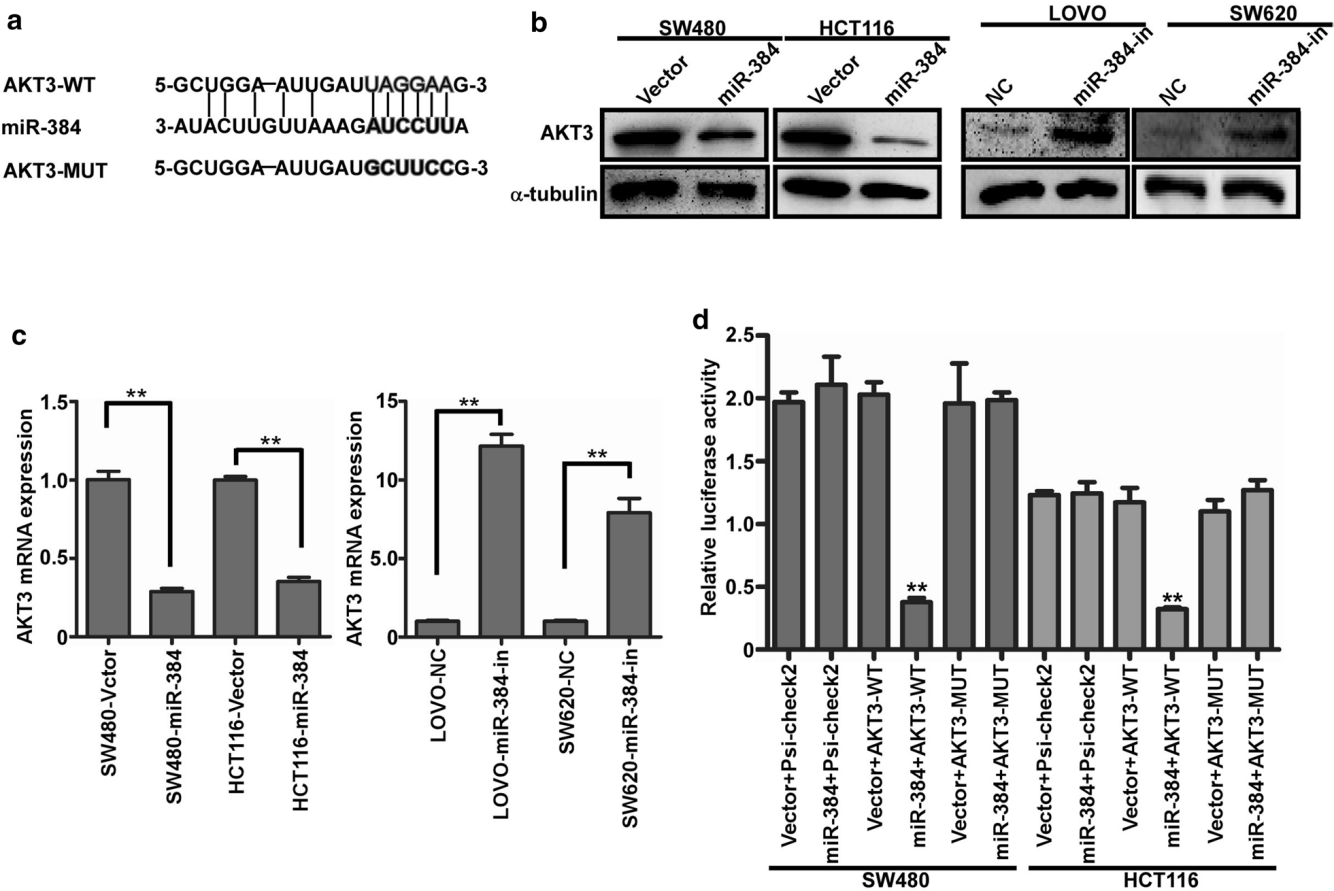
MiR-384 decreased AKT3 expression by directly binding to it’s 3′UTR in CRC. **a** Predicted miR-384 target sequences in the 3′UTRs of AKT3, and their mutants containing altered nucleotides in the 3′UTRs. **b** Western blot analysis of AKT3 in the indicated cells. **c** Real-time PCR analysis of AKT3 mRNA expression. **d** Luciferase assay analyses of the indicated cells transfected with the indicated reporters with miR-384 (error bars represent mean ± SD from three independent experiments; **p < 0.01)

The publicly available bioinformatic algorithms (TargetScan, miRada) were firstly used to analyze the target gene of miR-384. The results indicated that AKT3 was the theoretical target gens of miR-384 (Fig. 3a). Real-time PCR and western blot analyses showed that the mRNA and protein levels of AKT3 was significantly down-regulated in miR-384 over-expressed cells, whereas they were up-regulated with the inhibition of miR-384 (Fig. 4b, c). To confirm whether AKT3 was directly inhibited by miR-384, the dual-luciferase reporter system was performed. It was found that the co-expression of miR-384 markedly inhibited the Renilla luciferase reporter activity of the wild-type AKT3 3′UTR, but did not change the activity of the mutant 3′UTR constructs and their scramble vectors (Fig. 4d). The above results verified that AKT3 was the target gene of miR-384.

### Restoration the expression of AKT3 played an important role in miR-384-inhibited proliferation of CRC

**Fig. 5:**
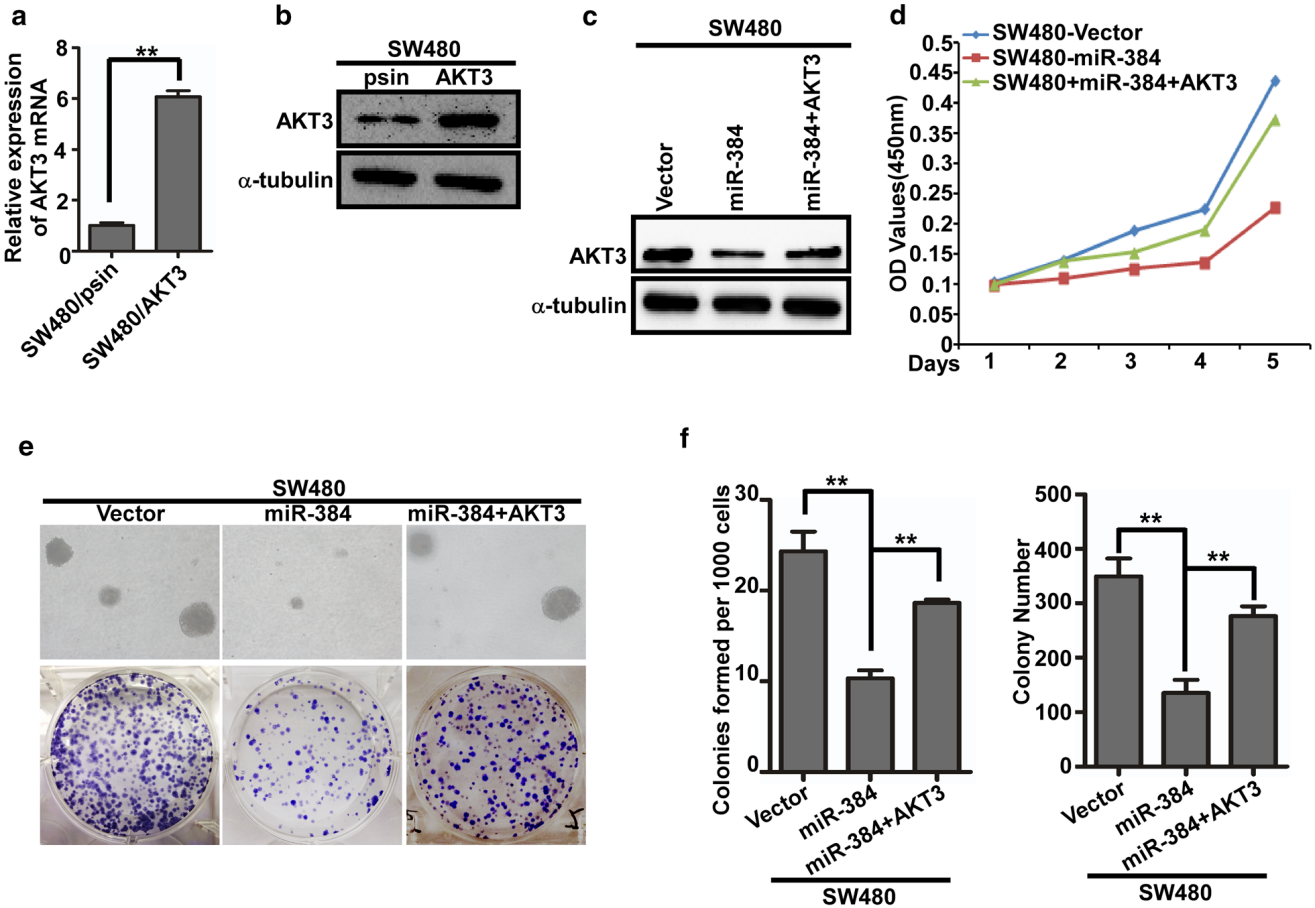
Restoration the expression of AKT3 played important roles in miR-384-inhibited proliferation of CRC. **a**–**c** AKT3 over-expression in SW480 cells by real-time PCR analysis or Western blot. **d**–**f** The proliferative ability of the indicated cells detected by MTT assays, colony formation assays and soft agar assays. Only cell colonies containing more than 50 cells were counted. Error bars represent mean ± SD from 3 independent experiments. **p < 0.01

To further confirm the role of miR-384 in the progression of CRC, we next restored the expression of AKT3 in SW480/miR-384 cells (Fig. 5c) by transfection of AKT3 ORF constructs without 3′UTRs, and observed their effects on proliferation. The results showed that the proliferative abilities of SW480/miR-384 cells increased with the restoration of AKT3 both in vitro (Fig. 5d–f). The in vivo tumorgenesis assay in nude mice showed the same trend (Additional file 1: Figure S1A, B). The above results confirmed that miR-384 inhibit the proliferation of CRC by targeting AKT3.

### The expression of miR-384 was negatively correlated with the expressions of AKT3 in CRC tissues

**Fig. 6:**
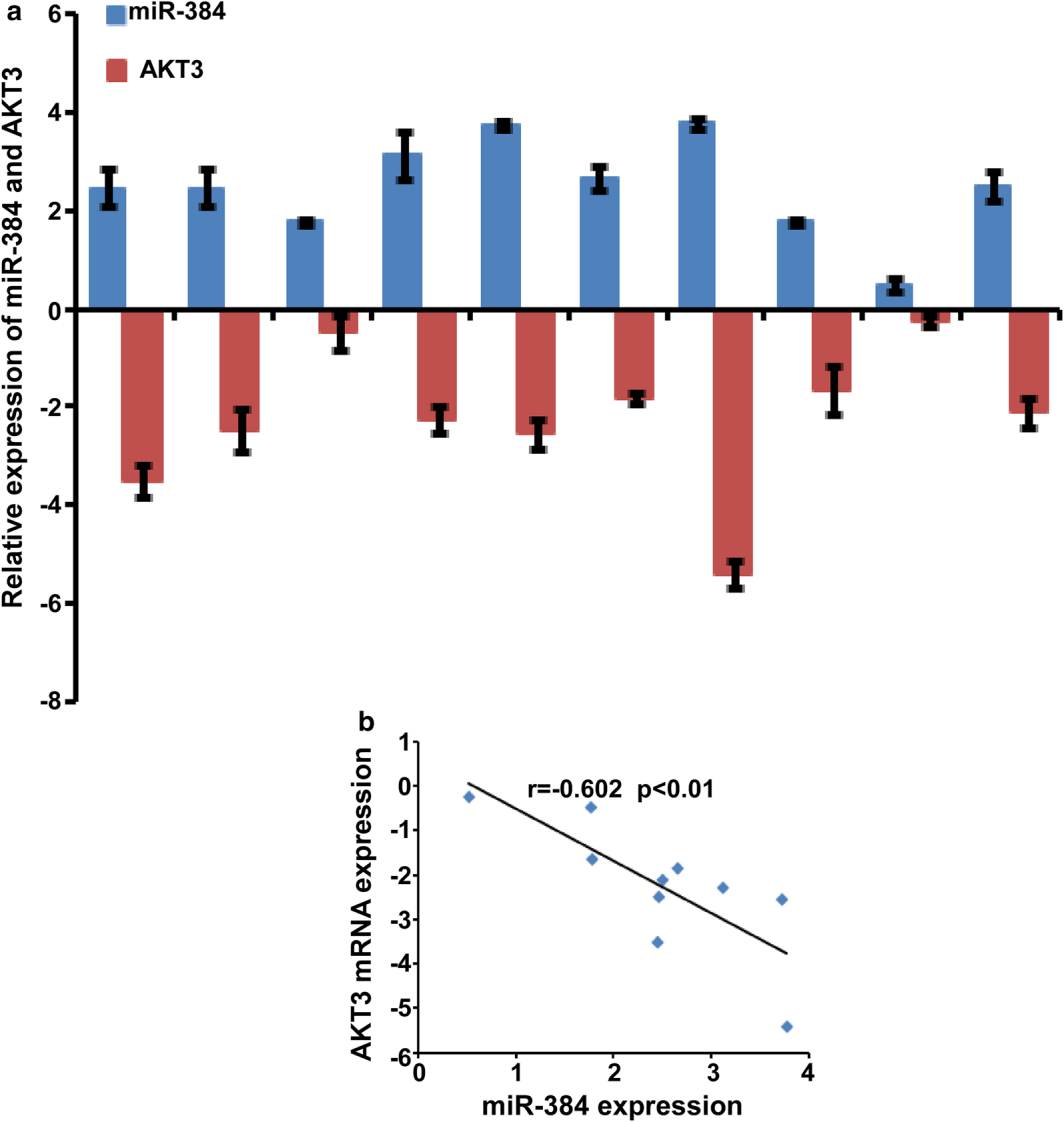
Restoration the expression of AKT3 played important roles in miR-384-inhibited proliferation of CRC. **a** The expression of miR-384 and AKT3 were detected by real-time PCR (ΔCт, n = 10). **b** Spearman correlation analyses of miR-384 expression and AKT3 mRNA expression

We detected the expression of AKT3 in 10 cases of CRC and their paired normal colorectal tissues and the data were normalized to 2 housekeeping genes (GAPDH and β-actin). The standard deviations normalized to GAPDH was lower than that of β-actin (Additional file 1: Figure S2A). Therefore, we used GAPDH for normalization of AKT3 expression analysis to further verify the correlation between the expression of miR-384 and AKT3 was analyzed in another 10 freshly collected CRC biopsies and paired normal tissues. It was showed that miR-384 was down-regulated in CRC tissues while AKT3 was up-regulated in CRC tissues (Fig. 6a). Spearman correlation analyses demonstrated that the expression of miR-384 expression was negatively correlated with the expressions of AKT3 (Fig. 6b).

## Discussion

MiRNAs are small regulatory molecules that negatively regulate their target gene by directly binding their mRNAs [17]. These small non-coding RNA molecules could function as oncogenes or tumor suppressors [18]. Accumulating evidences have verified that the dysregulation of miRNAs is closely related to the development and progression of cancer [19–22]. Recent studies have demonstrated that miRNAs play essential roles in the initiation and progression of CRC in recent studies [23, 24]. Up to now, the deregulation of miR-384 has only been observed in a few tumor types, suggesting its function as a cancer suppressor gene. For instance, a microarray showed that miR-384 was down-regulated in laryngeal carcinoma [25]. Another research implies mir-384 might play an important role in metastasis of melanoma by binding to the 3′UTR of HDAC3 [26]. Recently, it was reported that miR-384 exerted tumor-suppressive functions by binding to the 3′UTR of PIWIL4 in glioma [27]. However, it was not clear whether the dysregulation of miR-384 was associated with the proliferation of CRC. In our previous study, it has shown that with the restoration of KRAS and CDC42, the number of metastatic nodules in SW480 cells with miR-384 over-expression restored, too. But the volume of metastatic nodules could not be restored accordingly. This result suggested miR-384 might regulate the proliferation of CRC. Therefore, we focused on delineating the role and mechanism of miR-384 in CRC proliferation in the current study.

To explore the function of miR-384 in the proliferation of CRC, MTT, colony formation and soft agar assays were conducted. The results showed that the growth and proliferation of CRC cells were obviously inhibited by miR-384 over-expression in vitro. Moreover, the results of the tumourigenesis assay in nude mice verified that miR-384 significantly promoted the growth of CRC cells in vivo. Moreover, the inhibition of miR-384 significantly promoted the proliferation in vitro and the ability of tumor formation in vivo of the CRC cells. In conclusion, the results demonstrated that miR-384 could inhibit the proliferation of CRC. Therefore, it is crucial to elucidate the molecular mechanism underlying the inhibition role of miR-384 in CRC proliferation, which would contribute to providing potential therapeutic targets for CRC.

As we know, miRNAs functions through regulating their target genes by cleavaging their mRNA and inhibiting the synthesis of the protein. In this study, we selected AKT3 as a target gene of miR-384 via the publicly available bioinformatic algorithms analysis. AKT3 has been reported to be involved in cancer progression and function as an oncogene in many types of cancers by regulating the PIK3/AKT signal pathway [28–30]. Recently, it was found that the expression of AKT3 was upregulated in thyroid cancer and that the inhibition of AKT3 inhibited it’s proliferation [31]. In this study, we detected the expression of AKT3 in CRC and found that it was significantly upregulated. Notably, AKT3 has been found to be regulated by several miRNAs, such as miR-29a, miR-338-3p and miR-145 [32–34]. To further verify whether AKT3 was a target gene of miR-384 in CRC, luciferase reporter assay, qRT-PCR, and western blot were conducted. The results confirmed that AKT3 was a new target of miR-384. In addition, we found that the expression of AKT3 was upregulated in CRC tissues and was inversely correlated with miR-384 expression. Furthermore, it was found that the importance of AKT3 in mediating the effect of miR-384 was substantiated by the finding that AKT3 overexpression rescued the miR-384-mediated inhibitory effect on CRC cells.

## Conclusion

In brief, our findings confirmed that miR-384 suppressed the proliferation of CRC by directly targeting AKT3. Identification of the miR-384/AKT3 axis in CRC proliferation would contribute to better understanding of the molecular mechanisms underlying CRC and provide potential diagnostic and prognostic biomarker for CRC.

## Additional file

**Additional file 1.** Additional table and figures.

## Abbreviations

CRC: colorectal cancer
miR-384: microRNA 384
AKT3: AKT serine/threonine kinase 3
RT: reverse transcription
RT-qPCR: real time quantitative polymerase chain reaction
MTT: 3-(4,5-dimethylthiazol-z-yl)-2,5-diphenyltetrazolium bromide
DMSO: dimethyl sulphoxide
PVDF: polyvinylidene difluoride
