# Supplementary material for: MiR-384 inhibits the proliferation of colorectal cancer by targeting AKT3
Source: Cancer Cell Int. 2018 Sep 3;18:124. doi: 10.1186/s12935-018-0628-6 (PMC6122463; doi:10.1186/s12935-018-0628-6)
Supplement: Supplementary file 1 — Additional file 1. Additional table and figures. [file 12935_2018_628_MOESM1_ESM.doc]

**Additional file 1 Table:**

**Table S1.** Primer sequences used for target gene real-time PCR

| **Gene** | **Forward primer** | **Reverse primer** |
| --- | --- | --- |
| **AKT3** | ATACACGCAAATACACTCC | GACCACCTATGACATCTTC |
| **b-ACTIN** | CTGGAACGGTGAAGGTGACA | AAGGGACTTCCTGTAACAATGCA |
| **GAPDH** | GACTCATGACCACAGTCCATGC | AGAGGCAGGGATGATGTTCTG |

**Additional file 1 Figures and Figure legends:**


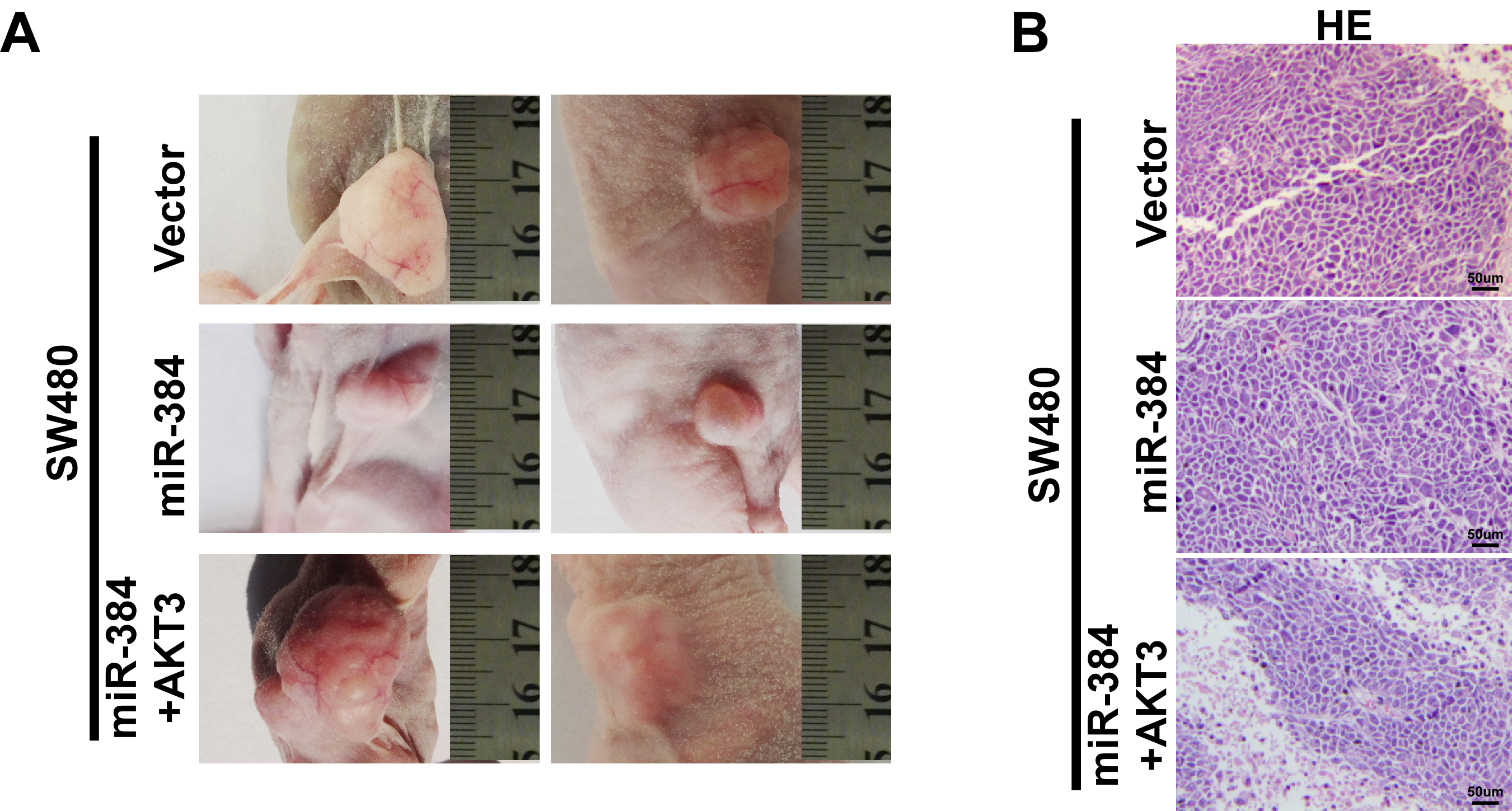


**Figure S1. Restoration the expression of AKT3 played important roles in miR-384-inhibited proliferation of CRC** (A) Gross images of the tumor in nude mice of the tumorigenesis assay. (B) Representative microscopical images of the tumor stained with H&E.


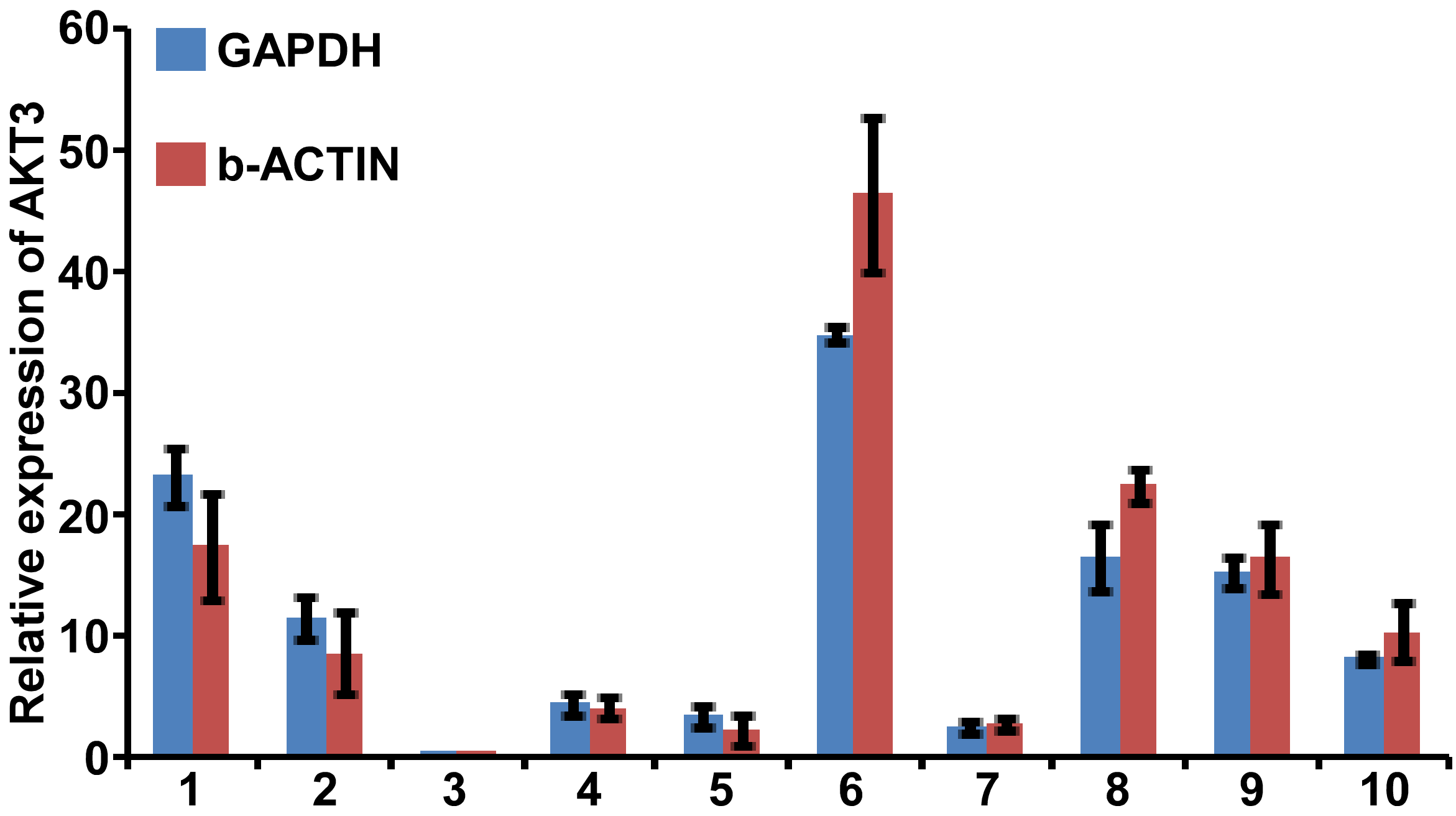


**Figure S2. The expression of AKT3 mRNA in CRC tissues by qRT-PCR (normalized to GAPDH and β-actin).**
